# Survey of young women's state of knowledge and perceptions about oral contraceptives in Germany

**DOI:** 10.1016/j.xagr.2022.100119

**Published:** 2022-10-07

**Authors:** Stella Juliane Vieth, Jamie Hartmann-Boyce, Nicolai Maass, Anant Jani

**Affiliations:** 1 Department for Continuing Education, University of Oxford, Oxford, United Kingdom; 2 Nuffield-Department of Primary Care Health Science, University of Oxford, Oxford, United Kingdom; 3 Klinik für Gynäkologie und Geburtshilfe, Campus Kiel, Universitätsklinikum Schleswig-Holstein, Kiel, Germany.

**Keywords:** doctor-patient relationship, gynecology, health education, oral contraceptives, transparency in healthcare

## Abstract

**BACKGROUND:**

In Germany, we see a decline in the use of the oral contraceptive pill. Although there have been studies showing a lack of knowledge about the mode of action of the pill and alternative methods, the number of German women who feel well informed about the pill increased over recent years. At the same time, a trend to increasingly cover negative aspects of oral contraception has emerged in German journalism and social media.

**OBJECTIVE:**

This study aimed to consider the relationship between the source of information about the pill, subjective and objective knowledge, and how their interaction influences perceptions of the pill.

**STUDY DESIGN:**

An online survey was conducted of 18- to 29-year-old women to test their objective and subjective knowledge, their perception of oral contraceptives, and their trust in gynecologists. The recruitment took place online and in gynecologic practices. The survey opened in September 2020 and closed in April 2021.

**RESULTS:**

A total of 2470 women completed the survey. The most common sources of information were the internet (80%), the gynecologist (47%), and friends and family (47%). Women reporting the internet as a source of information were more likely to have lower perception and trust rates, and less likely to overestimate their own knowledge. The findings suggest that school or university as a source of information has a positive effect on decision-making and general attitude toward information received by gynecologists about oral contraceptives. Those with higher confidence in their knowledge are likely to have a more positive attitude and higher levels of trust.

**CONCLUSION:**

A feeling of uncertainty, instead of fixed assumptions gathered from unsophisticated sources, affects perception regarding oral contraceptives and trust toward gynecologists negatively. Gynecologists and educators should hence increase efforts to meet potential needs for discussing uncertainties to prevent further loss of confidence.

**AJOG Global Reports at a Glance**

**Why was this study conducted?**

In Germany, we see a decline in the use of oral contraceptives in young women. Coincidentally, previous studies have reported uncertainty and a lack of information about the pill. This study aims to understand the available sources of information, young women's attitude toward and knowledge about the pill, and the driving forces of change in contraceptive behavior.

**Key findings**

Findings suggest that a woman's attitude toward the pill is associated with her source of information and how well informed she feels.

**What does this add to what is known?**

This study presents a direct comparison of subjective and objective knowledge, and hence allows for the understanding of the decisive part that self-estimation plays regarding attitude toward the pill and the information received from the gynecologist and/or other sources.

## Introduction

In Germany, oral contraceptives (“the pill”) are the most used method of contraception.^1^ Studies show high levels of uncertainty and hesitance in women concerning this topic,^1^, ^2^, ^3^ but the number of German women feeling well or very well informed about oral contraceptives has increased over the last 10 years.^1^ At the same time, a decline in the use of the oral contraceptive pill has been observed.^1^

Comments under scientific postings on social networks reveal high skepticism and loss of trust in medical caregivers when it comes to the pill. Thus, there are reasons to speculate that the recent decline in use is associated with trends in social media and media coverage. This may imply that although women feel better informed today, they may be influenced by unscientific journalism that prevents them from making informed decisions about birth control. This has the potential to lead to polarization and may hinder balanced consideration of risks and benefits for each individual woman. That might lead to both over- and underuse of contraceptive resources in women who might, if correctly informed, benefit from either the use or nonuse of the pill.

This study aimed to investigate how sources of information (SoI) influence the subjective and the actual state of knowledge about the pill in young German women, their perceptions of oral contraception (OC), and their trust in their gynecologist. Furthermore, we set out to examine associations between subjective and objective states of knowledge regarding the pill, the participants’ perception of the pill, and their trust in their gynecologist.

## Materials and Methods

JISC Online Survey was used to create and distribute a structured online, anonymous survey.^4^ A gynecologist provided feedback on the content of the questionnaire (Supplement A), which was also piloted among women meeting the inclusion criteria.

Potential participants were approached during visits to their gynecologist. The inclusion criteria were met by biological women, aged between 18 and 29 years, who did not have any indication for taking the pill other than pregnancy prevention. Participants were asked to give informed consent and declare to be over 18 years old.

To gain a wide spectrum of thoughts about the contraceptive pill from women of different socioeconomic backgrounds, various gynecologic practices in Berlin and the northern area of Germany were asked to participate. Furthermore, the flyer and link were posted on social media platforms via the official account of the cooperating university clinic in Kiel, Germany.

A random sample of 385 women was needed for this study. Details on sample size calculation can be found in Supplement B.1.

Management of the data complied with the requirements of the General Data Protection Regulation and the Data Protection Act 2018. Ethical applications were approved by the Departmental Research Ethics Committee at the University of Oxford (ref: EQ C1A 20 037) and by the ethics committee at the University of Kiel (ref: D53/120).

The survey opened in September 2020 and closed on April 1, 2021. Participation in this study was voluntary and no incentives were offered to the participants.

### Analysis

The questionnaire contained a knowledge quiz with a maximum achievable score of 29.5 points. Former studies revealed that the perception about the pill's efficacy in pregnancy prevention, the uncertainty about its negative effects on physical and psychological health, and its hormonal ingredients play an important role when deciding for a contraceptive method.^1^, ^2^, ^3^^,^^5^ Therefore, knowledge about mode of action, safety of pregnancy prevention, and side effects of the pill was tested. The test was evaluated by a gynecologist and piloted among women meeting the inclusion criteria.

The participants were categorized into 1 of 5 groups on the basis of their objective knowledge score. Furthermore, participants were asked to estimate their subjective knowledge (Supplement B.2), which was disaggregated across 5 levels of knowledge.

The accuracy of self-estimation was measured as the direct comparison of the total subjective knowledge score and the achieved knowledge score. The participants were grouped into 1 of 3 categories: “Underestimation,” “Accurate estimation,” and “Overestimation.”

Multinomial logistic regression analysis and a univariate logistic regression analysis were conducted using IBM SPSS Statistics, Version 26.0 (IBM Corp, Armonk, NY).^6^ Unadjusted results and results adjusted for birth year and educational achievement were reported. A *P* value of <.05 was used to determine statistical significance.

Nine questions and the feedback section allowed for free-text answers. None of the free-text answers were included in the analyses, but they were used to specify the context and were taken into consideration during the interpretation of the results.

Unless stated otherwise, the reported odds ratios (ORs) were adjusted for age and educational background.

## Results

The recruitment in German practices started on September 15, 2020 and continued until the survey closure on April 1, 2021. There was an immediate drop-off of 41.6% after the information and consent page from 5136 to 3001 participants (Supplement C.1). Subsequently, a steady decline was reported, with no specific question leading to termination in a high proportion of participants. A total of 2470 women completed the survey.

### Demographics

The mean age was 23.91 (standard deviation, ±3.27) years (Supplement C.2). Most participants reported the German general university entrance qualification called “Abitur” as their current highest educational achievement (38.5%). Most of the women surveyed were unmarried (89.7%), but 73.2% (n=1808) stated that they were in a relationship; 88.9% (n=2195) reported being heterosexual (Table 1).

**Table 1:** Demographic data, frequency, and proportions

Demographic data	Frequency	Percentage
Current highest educational achievement		
Lower secondary school diploma	11	0.4
Intermediate secondary school leaving certificate	97	3.9
College entrance qualification	140	5.7
Abitur	951	38.5
Postgraduate degree	449	18.2
University degree	812	32.9
No statement	10	0.4
Total	2470	100
Current marital status		
Married	154	6.2
Unmarried	2215	89.7
No statement	101	4.1
Total	2470	100
Are you in a relationship?		
Yes	1808	73.2
No	639	25.9
No statement	23	0.9
Total	2470	100
Sexual orientation		
Asexual	10	0.4
Bisexual	211	8.5
Heterosexual	2195	88.9
Homosexual	12	0.5
Other	18	0.7
No statement	24	1
Total	2470	100

Vieth. Survey of young women's state of knowledge and perceptions about oral contraceptives in Germany. Am J Obstet Gynecol Glob Rep 2022.

### Experience with and perceptions of the pill

Of the women in the sample, 92.7% (n=2290) had taken the pill at some point in their life; 64.5% (n=1480) of those who had ever taken the pill stopped doing so (Supplement E.1). The most common reasons for discontinuation were concerns about long-term effects (66.1%; n=979) and experience of side effects (54.8%; n=813) (Table 2). The SoI most reported by the women participating in this study was the internet (79.8%) (Table 3).

**Table 2:** Rationale for discontinuation of oral contraception

Reasons for discontinuation (multianswer)						
Concerns about long-term effects (%)	Experience of side effects (%)		No longer need/wish to prevent pregnancy (%)	Other reasons (%)	No statement (%)	
979 (66)	813 (54.8)		222 (15)	123 (8.3)	5 (0.3)	
Other reasons:						
Headline		Example (translation)				N
Hormones		“I wanted to get to know my body independently from the influence of artificial hormones.”				45
Daily intake		“I did not want to think about it each day.”				18
Change of method		“I wanted to try a different method.”				29
Side effects or suspected side effects		“I felt emotionally controlled by an external source.” “I just did not feel fine anymore.” “My libido decreased.”				30
Medical contraindications		“Migraine with aura”; “prior thrombotic events”				15
Ecological reasons		“I am worried about hormones in water.”				3
By accident		“I forgot to take enough packages with me on a trip.”				2
Others		“I got pregnant despite the pill.” “I did not feel well informed due to the information I got from my gynecologist.”				5

Vieth. Survey of young women's state of knowledge and perceptions about oral contraceptives in Germany. Am J Obstet Gynecol Glob Rep 2022.

**Table 3:** Frequency and proportions: source of information; “Would you recommend your daughter to take the pill if she was in the appropriate age now?”; subjective knowledge (excluding “I don't know” or “no statement”)

Source of information (multianswer)	Frequency	Percentage
Gynecologist	1892	76.6
Friends and family	1155	46.8
School or university	335	13.6
Internet and social media	1972	79.8
Books and papers	567	23.0
No statement	6	0.2
Other sources	28	1.1
Total	5955	241.1
Would you recommend your daughter to take the pill if she was in the appropriate age now?		
Yes.	126	5.1
Rather yes.	488	19.8
Rather no.	816	33.0
No.	687	27.8
I don't know.	340	13.8
No statement.	13	0.5
Total	2470	100
Subjective Knowledge (excluding “I don't know” and “No statement”)		
Very high	237	9.6
High	1104	44.6
Medium	906	36.6
Low	156	6.3
Very low	18	0.7
Total	2421	98.0
How much do you trust the information about oral contraceptives given to you by your gynecologist?		
Completely	256	10,4
Enough	657	26,6
Not in all respects	1002	40,6
Not enough	405	16,4
Not at all	120	4,9
No statement	30	1,2
Total	2470	100

Vieth. Survey of young women's state of knowledge and perceptions about oral contraceptives in Germany. Am J Obstet Gynecol Glob Rep 2022.

Of the women who had ever taken the pill, 35.4% (n=811) were still taking it at the time of the survey. Within this group, 19.2% (n=156) reported being very satisfied, and 40.6% (n=329) reported being satisfied. Another 39.5% (n=321) stated that they were unsatisfied and/or currently thinking about stopping or changing to another method; 0.6% (n=5) chose not to give a statement (Supplement E.1). The most common cited reasons for considering discontinuation were concerns about the long-term effects (Table 4).

**Table 4:** Perception and rationale for perception; frequency and proportion

I am currently thinking about stopping/changing to another method because… (multianswer)	Frequency	Percentage
… I no longer want/need to prevent pregnancy.	8	1.4
… I am experiencing side effects.	180	32.5
… I worry about my fertility.	99	17.9
… I am worried about the long-term effects.	236	42.6
No statement.	7	1.3
Other reasons.	24	4.3
Total:	554	100
How did your perception of the pill change since you first heard about it?		
Not at all.	295	11.9
I have a more positive perception of the pill than I had before.	44	1.8
I have a more negative perception of the pill than I had before.	2099	85.0
No statement	32	1.3
Total	2470	100
If you stated a change in perception in the previous question, what are the reasons for that change? (multianswer)		
Own experience	1454	58.9
Experiences and stories shared by friends and family	1283	51.9
Journalism and public media	1091	44.2
Information found in advice literature	449	18.2
Information found on the internet	1226	49.6
Information given to me by my gynecologist	164	6.6
I don't know.	7	0.3
No statement	10	0.4
Others	19	0.8
Total	5703	230

Vieth. Survey of young women's state of knowledge and perceptions about oral contraceptives in Germany. Am J Obstet Gynecol Glob Rep 2022.

Twelve percent (n=259) of the women reported no change, 85.0% (n=2099) reported a more negative perception, and 1.8% (n=44) reported a more positive perception about the pill relative to their perception in the past. The most common reported reasons for this change were personal experience (58.9%), experiences and stories shared by family and friends (51.9%), and information found on the internet (49.6%) (Table 4).

### Knowledge about and perceptions of the pill

With regard to knowledge about the pill, 67.2% (n=1659) of the women stated they would like to be better informed, 18.5% (n=456) indicated they did not feel a need for further information, and 12.1% (n=298) stated they did not know (Supplement E.2). When asked about their subjective knowledge about mode of action, safety, and side effects of the pill respectively, the women felt the least informed about side effects (Supplement E.3). The total estimate of the subjective knowledge reported by women can be found in Table 3.

**Figure 1:**
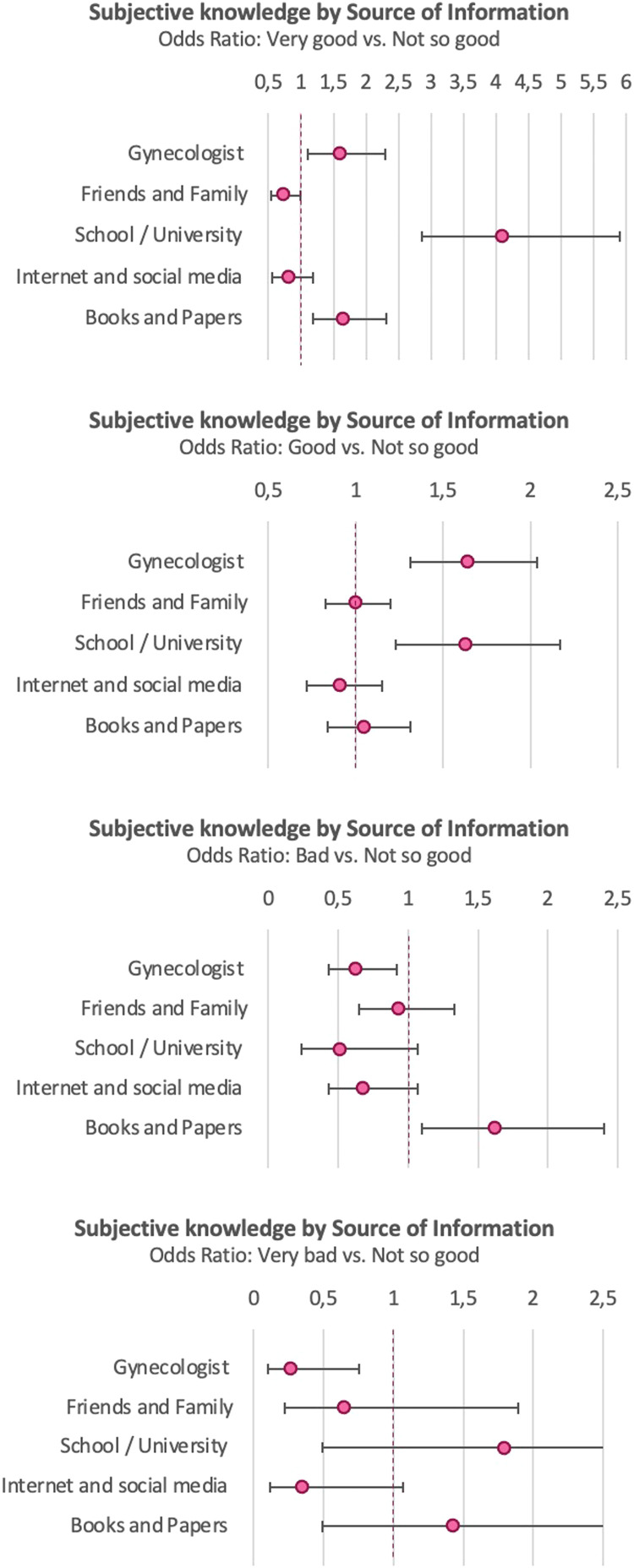
Subjective knowledge by source of information Odds ratio with 95% confidence interval. Reference category: “Not so good.” The subjective state of knowledge in women seems to depend on their source of information. Participants receiving their information from their gynecologist or in school or university were more likely to feel very well or well informed compared with those not using the respective sources.

Participants receiving their information from their gynecologist, in school or university, or from books and papers were more likely to feel very well informed (OR, 1.59; confidence interval [CI], 1.10–2.30; OR, 4.11; CI, 2.86–5.90; and OR, 1.65; CI, 1.18–2.31, respectively) compared with those not receiving their information from the respective sources. Those reporting friends and family as their SoI were less likely to feel very well informed (OR, 0.73; CI, 0.54–1.00). Women reporting their gynecologist or school and university as their SoI were also more likely to feel well informed (OR, 1.64; CI, 1.31–2.04; and OR, 1.63; CI, 1.23–2.17, respectively) and less likely to feel badly (OR, 0.63; CI, 0.43–0.92; and OR, 0.51; CI, 0.24–1.07) or very badly (OR, 0.27; CI, 0.10–0.75; and OR, 0.35; CI, 0.12–1.07) informed. Women reporting books and papers as their SoI were more likely to feel badly informed compared with those not using this SoI (OR, 1.62; CI, 1.10–2.40) (Figure 1; Supplement D.1).

*Vieth. Survey of young women's state of knowledge and perceptions about oral contraceptives in Germany. Am J Obstet Gynecol Glob Rep 2022*.

Women receiving their information from their gynecologist and those receiving their information in school or university were less likely to report a change toward greater negative perception of the pill (OR, 0.62; CI, 0.44–0.87; and OR, 0.54; CI, 0.39–0.74) compared with those using other SoI. Those informing themselves via the internet and social media were more likely to have become more critical of the pill (OR, 2.01; CI, 1.51–2.69) (Supplement D.4).

An increase in the final score was associated with a decrease in the odds of reporting to be more critical relative to the past (OR, 0.96; CI, 0.93–1.00) (Supplement D.7). Women with very high or high subjective knowledge were less likely to report a change toward either less (OR, 0.14; CI, 0.02–0.76; and OR, 0.19; CI, 0.04–0.90) or more critical (OR, 0.06; CI, 0.02–0.18; and OR, 0.12; CI, 0.04–0.32) perception compared with those with low or very low subjective knowledge (Supplement D.10).

When asked whether they would recommend the pill as a contraceptive method to their hypothetical daughter, most women responded negatively: 5.1% responded with “yes,” 19.8% “rather yes,” 33.0% “rather no,” and 27.8% “no”; 13.8% of the participants reported they did not know (Supplement E.4). Increase in the knowledge test score was associated with an increase in odds for stating “mostly yes” (OR, 1.07; CI, 1.02–1.11) (Supplement D.9).

Compared with the group with low or very low subjective knowledge, there were significantly higher odds for answering “yes” than “I don't know” to the above question among women reporting very high or high subjective knowledge (OR, 10.95; CI, 1.36–88.06; and OR, 8.29; CI, 1.07–64.06). Women with high subjective knowledge were also more likely to answer “mostly yes” (OR, 3.96; CI, 1.64–9.58). Those with very high subjective knowledge were on average less likely to answer “rather no” (OR, 0.40; CI, 0.20–0.80) or “no” (OR, 0.28; CI, 0.14–0.54). Women with high subjective knowledge and those who estimated their knowledge as average were also less likely to respond “no” compared with those with low or very low subjective knowledge (OR, 0.25; CI, 0.14–0.45; and OR, 0.39; CI, 0.22–0.69) (Supplement D.12).

### Measured state of knowledge

**Figure 2:**
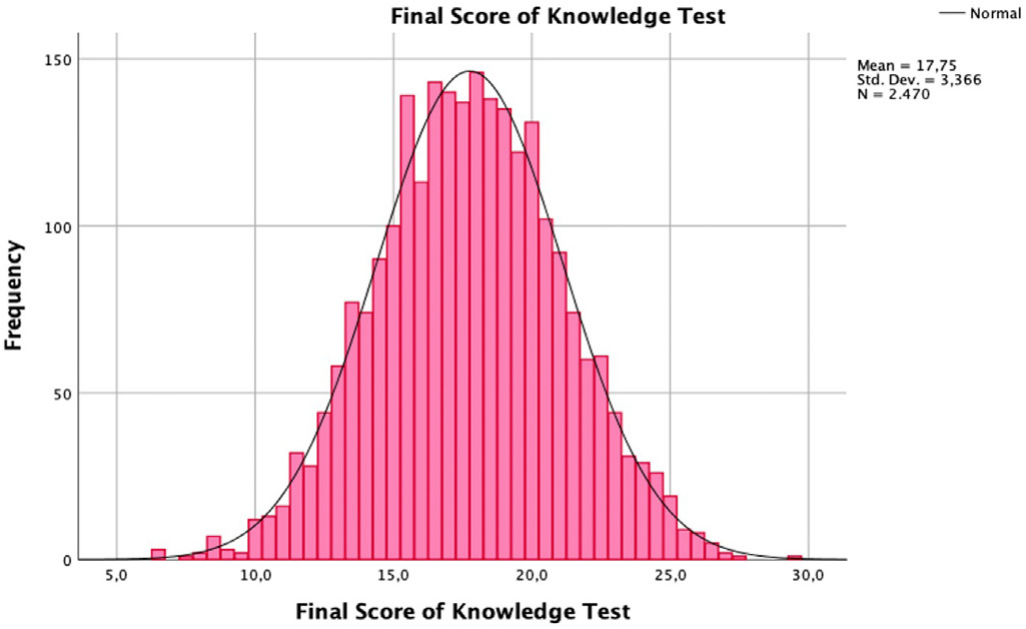
Diagram of final score of knowledge test, frequency The mean knowledge test score was 17.75 points, with a standard deviation of 3.37. The participant with the lowest number of points achieved 6.5 points, and the highest score achieved was the maximum score with 29.5 points.

**Figure 3:**
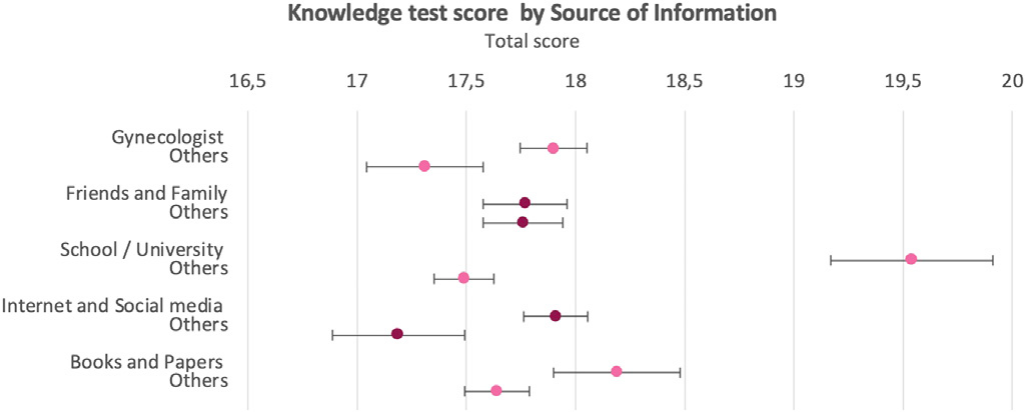
Knowledge test score by source of information Average score with 95% confidence interval. Four of the 5 different sources of information led to an increase in average knowledge test score. These findings were significant for gynecologists (B=0.6; SE, 0.16), school and university (B=2.05; SE, 0.19), internet and social media (B=0.81; SE, 0.17), and books and papers (B=0.5; SE, 0.16) as sources of information when compared with not using these sources of information. *SE*, standard error.

The mean knowledge test score was 17.75 points, with a standard deviation of 3.37 (Figure 2). Four of the 5 SoI were associated with an increase in average knowledge test scores. Those findings were statistically significant for gynecologist (B=0.6; standard error [SE], 0.16), school and university (B=2.05; SE, 0.19), internet and social media (B=0.81; SE, 0.17), and books and papers (B, 0.5; SE, 0.16) as SoI when compared with not using these SoI (Figure 3; Supplement D.2).

*Vieth. Survey of young women's state of knowledge and perceptions about oral contraceptives in Germany. Am J Obstet Gynecol Glob Rep 2022*.

*Vieth. Survey of young women's state of knowledge and perceptions about oral contraceptives in Germany. Am J Obstet Gynecol Glob Rep 2022*.

**Figure 4:**
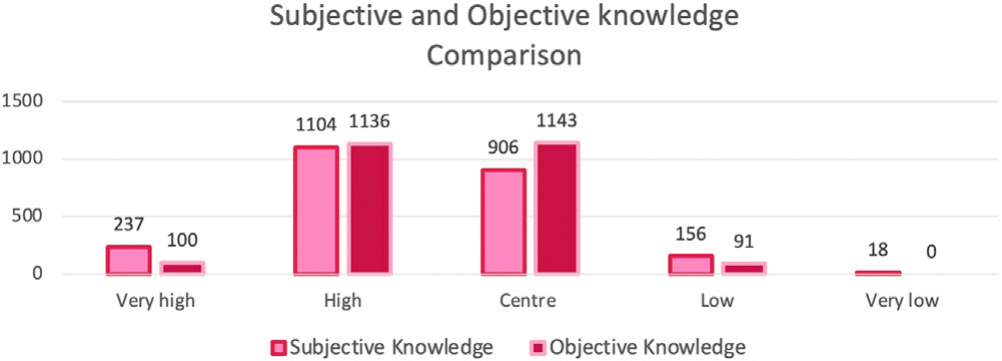
Diagram of comparison of subjective and objective knowledge, frequency The differences in frequency for each group show that 237 women estimated their knowledge to be very high, whereas only 100 reached this category. There were 32 women more than subjectively estimated achieving high knowledge scores, and 237 more in the center group. Less women than subjectively estimated had a low or very low level of knowledge (n=65; 18).

The participants were sorted into 5 groups on the basis of their achieved score. The differences in frequency for each group showed that 237 women estimated their knowledge to be very high, whereas only 100 reached this category; 44.6% gave a correct estimation of their state of knowledge, and 29.8% overestimated and 23.6% underestimated their knowledge (Figure 4).

*Vieth. Survey of young women's state of knowledge and perceptions about oral contraceptives in Germany. Am J Obstet Gynecol Glob Rep 2022*.

For women using the internet and social media for information, the relative odds of overestimating their knowledge were decreased by a factor of 0.66 (OR, 0.66; CI, 0.52–0.84). Women receiving their information from their gynecologist were statistically significantly less likely to underestimate their knowledge compared with women not receiving their information from their gynecologist (OR, 0.69; CI, 0.55–0.88) (Supplement D.3).

### Trust in gynecologist

**Figure 5:**
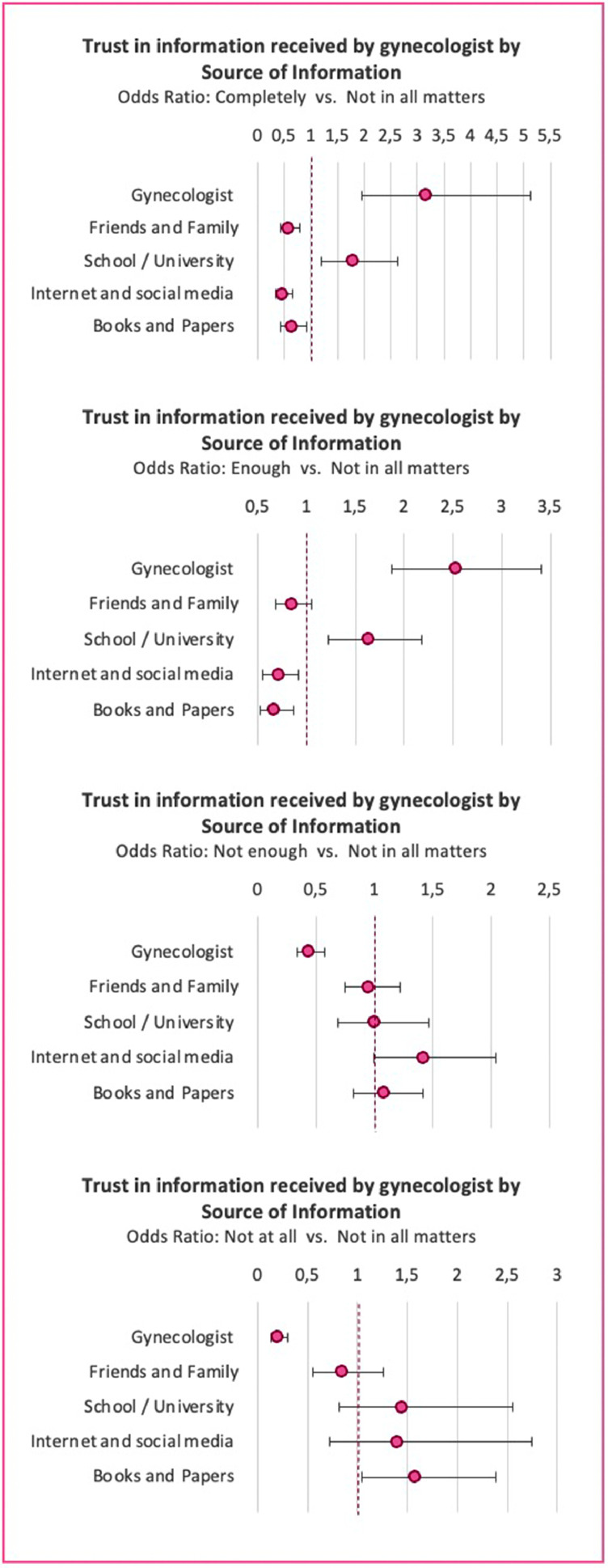
Trust in information received by gynecologist by source of information OR with 95% CI. Reference category: “Not in all matters.” Women who reported receiving their information from their gynecologist or at school/university were the only 2 groups that were more likely to “completely” trust their gynecologist when compared with women using other sources of information (OR, 3.16; 95% CI, 1.95–5.12; and OR, 1.78; 95% CI, 1.20–2.64). All other groups were statistically significantly less likely to completely trust their gynecologist than to trust them “not in all matters” at the 5% significance level. *CI*, confidence interval; *OR*, odds ratio.

The largest group of women stated their level of trust in the information provided by their gynecologist as “not in all respects” (40.6%) (Table 3). Women receiving their information from their gynecologist or at school or university were the only 2 groups that were more likely to “completely” trust their gynecologist when compared with women using other SoI (OR, 3.16; CI, 1.95–5.12; and OR, 1.78; CI, 1.20–2.64). All other groups were statistically significantly less likely to completely trust their gynecologist than to trust them “not in all matters,” at a 5% significance level. The groups informing themselves via friends and family, internet and social media, or books and papers were also less likely to state that they trusted their gynecologists “enough.” However, that was only statistically significant for the latter 2 groups (OR, 0.72; CI, 0.55–0.92); and OR, 0.67; CI, 0.52–0.87). The groups reporting their gynecologist or school or university as their SoI were again more likely to report trusting their gynecologist “enough” when compared with women using other informative methods (OR, 2.53; CI, 1.87–3.41; and OR, 1.63; CI, 1.22–2.18, respectively) (Figure 5; Supplement D.5).

*Vieth. Survey of young women's state of knowledge and perceptions about oral contraceptives in Germany. Am J Obstet Gynecol Glob Rep 2022*.

An increase in the final score led to a statistically significant increase in the odds of trusting their gynecologist's information “completely” (OR, 1.10; CI, 1.06–1.15) or “enough” (OR, 1.06; CI, 1.03–1.10) (Supplement D.8).

Women with very high or high subjective knowledge were more likely to trust their gynecologist “completely” (OR, 12.28; CI, 4.97–30.32; and OR, 4.38; CI, 1.87–10.30) or trust them “enough” (OR, 10.09; CI, 4.51–22.59) compared with those with low or very low subjective knowledge. They were also statistically significantly less likely to state “not enough” (OR, 0.55; CI, 0.32–0.97; and OR, 0.32; CI, 0.22–0.48). Those with high subjective knowledge were also less likely to state trusting them “not at all” (OR, 0.20; CI, 0.11–0.37). Women with medium subjective knowledge also had a lower OR of stating “not enough” (OR, 0.65; CI, 0.44–0.95) and “not at all” (OR, 0.46; CI, 0.27–0.81) compared with those with low or very low subjective knowledge (Supplement D.11).

The aspect about which most of the women felt the least comfortable were long-term effects on fertility (50.4%), followed by common side effects (48.5%) and long-term effects on cancer risk (39.8%) (Supplement E.5).

## Discussion

This study suggests a desire for more information about OCs, a considerable uncertainty regarding the side effects, a shift toward a more critical perception of OCs, and a lack of trust regarding the information provided by their gynecologist among German women aged between 18 and 29 years. The importance of the internet as a SoI seems to be increasing, and its use is associated with a higher degree of skepticism about information about OCs provided by gynecologists.

The most common reason for discontinuation of OCs seems to be concerns and uncertainties regarding short- and long-term effects of the hormonal ingredients. What affects the perception toward the pill negatively are not fixed assumptions gathered from unsophisticated sources, but a feeling of unease and uncertainty resulting from a lack of easily accessible evidence-based information. These uncertainties seem to harm trust in relationships between patients and doctors. Information gathered from school or university seems to have a positive effect on women's trust in gynecologists, their perception of the pill, and their level of knowledge. Increasing quality and accessibility of sexual education in schools is hence a valuable means of supporting informed decision-making and improving patient–doctor relationships. Furthermore, open communication and clarification of evidence-based findings may help reestablish trust between patients and their gynecologists, but further research is needed to investigate this.

### Strengths and limitations

The first limitation concerns the generalizability of the results because of the use of a convenience sample. Efforts to reduce the total survey error were made during the design of this study.

Using social media in the recruitment strategy may have distorted the results regarding the use of internet as an SoI. However, with respect to the age of the audience, there is reason to suspect that the internet is likely to be ever-present independently of the use of social media.

The inability to calculate the response rate presents another limitation of the study. The respective numbers of participants recruited through flyers and online posts cannot be determined. Thus, the presence of nonresponse error owing to total nonresponders cannot be excluded.

Multiple testing is also a limitation of this study given that the high number of tests conducted might have increased the findings of spurious but statistically significant associations. Hence, the findings of this study are to be interpreted as the results of explorative research and should not be understood as establishing causality or generalizable results.

## Conclusion

Findings of this explorative study indicate that women's attitudes toward OCs and their gynecologists’ information on OCs depend on their SoI. Women receiving their information from friends and family, the internet, or books and papers seem to have less positive perceptions of OCs and less trust in their gynecologist. They were also less likely to feel well informed compared with women receiving information in school or university or from their gynecologists. The only association between SoI and accuracy of self-estimation found in this sample was that those informing themselves via the internet were less likely to overestimate their knowledge, whereas those reporting their gynecologist as SoI were less likely to underestimate it. Both groups achieved a very similar knowledge score. This suggests a high degree of confidence among those receiving their information from their gynecologist, and less certainty among those informing themselves via the internet.

Results showed that individuals with higher objective knowledge were likely to have a more positive attitude toward the pill and information provided by their gynecologists. The increasing importance of the internet as an SoI should be acknowledged by healthcare professionals and educators. Strategies to increase the quality of information available on social media and the internet should be considered. Although women using these SoI seem to be well informed, they show exaggerated uncertainties regarding side effects and long-term health concerns. The discussion of oral contraceptives seems to have the potential to harm the trust between women and their gynecologists. Schools and universities should be acknowledged as SoI with high potential to improve sexual health literacy in women, and further efforts should be undertaken to improve access to and quality of healthcare education in schools. Furthermore, gynecologists should increase efforts to thoroughly solve any ambiguities and meet potential needs for discussing uncertainties to prevent further loss of confidence.

### Implications for further studies

Further studies should be conducted to learn about the kind of teaching programs and units in schools and universities that seem to help in establishing trust in medical science and its representatives. Furthermore, the association between perceptions of the pill and trust in gynecologists should be further studied to facilitate physicians’ efforts to stabilize their relationship with skeptical patients. Another subject of further studies could be which websites are used to obtain information about OCs, and what strategies could be used to limit the amount of inaccurate information. Learning about common information-seeking behaviors on the internet could enable women to access evidence-based findings and reduce uncertainty about OCs, hence facilitating informed contraceptive decision-making.

## Appendix Supplementary materials

Image, application 1

Image, application 2

Image, application 3

Image, application 4

Image, application 5
