# Supplementary material for: Survey of young women's state of knowledge and perceptions about oral contraceptives in Germany
Source: AJOG Glob Rep. 2022 Oct 7;2(4):100119. doi: 10.1016/j.xagr.2022.100119 (PMC9633744; doi:10.1016/j.xagr.2022.100119)
Supplement: Supplementary file 1 [file mmc1.docx]

Supplement A

**Questionnaire, Multiple choice**

**Exit button available on each page**

**Q3:** When are you born?

- - Drop-down list “year of birth”

**Q4:** What is your current highest grade of education?

- - Hauptschulabschluss (secondary modern school qualification)
  - Mittlerer Schulabschluss (high-school diploma)
  - Fachhochschulreife
  - Abitur (A-level)
  - Abgeschlossene Berufsausbildung / Examen (professional training)
  - Abgeschlossenes Studium (study degree)
  - No statement

**Q5:** Family status

- - married
  - unmarried
  - no statement

**Q6**: Are you currently in a relationship?

- - Yes
  - No
  - No statement

**Q7:** Sexual orientation

- - Asexual
  - Bisexual
  - Heterosexual
  - Homosexual
  - Other
  - No statement

**Q8**: Have you ever been taking the pill?

- - **Yes**

If yes: **Q9**  -> When did you first start taking the pill? When I was…

- - - - <16 years old.
      - 16-18 years old.
      - 18-20 years old.
      - >20 years old.
      - I don’t know
      - No statement

If yes:  **Q10 ->** Are you still taking the pill

- - - I used to take the pill, but I stopped

**->Q11**

- - - - Because I no longer wanted / needed to prevent pregnancy.
      - Because I was experiencing side effects.
      - Because I was worried about my fertility.
      - Because of other reasons.
      - No statement
    - I am currently still taking the pill

**->Q12**

- - - - … and am very satisfied with it.
      - … and am not very satisfied with it.
      - … and am currently thinking about stopping / changing to another method

->**Q15**

- - - - - Because I no longer want / need to prevent pregnancy
        - Because I’m experiencing side effects
        - Because I’m worried about my fertility
        - Because of other reasons
        - No statement
      - No statement
  - **No,** (Q8)

If no -> **Q13**

- - - I’ve never and it’s unlikely that I will ever start taking the pill.

-> **Q14**

- - - - Because I’ve heard many bad things about it.
      - Because I’m using a different contraceptive method.
      - Because of other reasons.
      - No statement
    - No, but it’s possible I will start someday.
    - No statement
  - No statement (Q8)

**Q16** Where are you getting your information about the pill?

- - My gynecologist
  - Family and Friends
  - School or Education
  - Internet or social media
  - Books and Journalism
  - Other
  - No statement

**Q17** Who has first introduced the topic of birth control to you?

- - Friends
  - School or Education
  - Gynecologist
  - Parents
  - Partner
  - I don’t remember
  - Other
  - No statement

Part 1: assessment of knowledge (4 Questions)

**Q18** Would you like to be better informed about oral contraceptive methods?

- - Yes
  - No
  - I don’t know
  - No statement

**Q19** How well do you feel informed about the mode of action of oral contraception?

- - Very well
  - Well
  - Poorly
  - Very poorly
  - I don’t know
  - No statement

**Q20** How well do you feel informed about the safety of oral contraceptives?

- - Very well
  - Well
  - Poorly
  - Very poorly
  - I don’t know
  - No statement

**Q21** How well do you feel informed about the side effects caused by oral contraceptives?

- - Very well
  - Well
  - Poorly
  - Very poorly
  - I don’t know
  - No statement

Part 2: Test your knowledge! 8 Questions

**Q22** What are possible methods of oral contraceptives preventing pregnancies? *Select all that apply* (1p. per correct / false answer identified as such)

- - Prevention of ovulation
  - Thickening of mucus
  - Killing of the sperm
  - Thinning the endometrium (inner line of uterus)
  - I don’t know

**Q23** What are the different options currently on the market? *Select all that apply* (1p. per correct / false answer identified as such)

- - Pills containing a combination of progesterone and estrogen
  - Pills containing a combination of testosterone and estrogens
  - Pills only containing estrogens
  - Pills only containing testosterone
  - Pills only containing progesterone
  - I don’t know

**Q24** Please select the options you would feel comfortable to explain to a friend

- - Monophasic pills
  - Multiphasic pills
  - Extended-cycle pills
  - None of the above
  - I don’t know

**Q25** What are common side effects of the pill? *Select all that apply* (0.5p. per correct / false answer identified as such)

- - Intermenstrual spotting
  - Nausea
  - Breast tenderness
  - Diarrhea
  - Headaches and migraine
  - Weight gain
  - Mood changes
  - Increased libido
  - Missed period
  - Decreased libido
  - Vaginal discharge
  - Changes in motoric abilities
  - Changes to eyesight for those using contact lenses
  - Other: _______
  - I don’t know

**Q26** Imagine Anna is currently preventing pregnancy with the Oral contraceptive pill. Yesterday, she forgot to take the pill. Which of the following statements are true? (2 pp. per correct answer)

- - Birth control won’t be effective for the rest of the day.
  - Birth control won’t be effective for 30 days from now on.
  - Birth control won’t be effective for the rest of the menstrual cycle.
  - None of the above are true
  - I don’t know

**Q27** Imagine Anna is currently preventing pregnancy with the Oral contraceptive pill. Yesterday, she forgot to take the pill. Which of the following statements are true? (2 pp. per correct answer)

- - That depends on how long Anna has been taking the pill before that incidence.
  - It is still safe, if this is the last pill in the box.
  - Birth control will still be effective, if Anna takes the pill today.
  - Birth control will still be effective, even if Anna forgets to take the pill again today.
  - None of the above
  - I don’t know

**Q28** Which of the following statements are true? *Select all that apply* (1p. per correct / false answer identified as such)

- - Oral contraceptives can have a positive effect on menstruation cycle
  - Oral contraceptives can have a positive effect on your mood
  - Oral contraceptives increase the risk of breast cancer
  - Oral contraceptives can decrease the risk of ovarian cancer
  - Oral contraception brands can be switched to alleviate side effects
  - I don’t know

**Q29** The risk of experiencing a blood clot in women taking the pill. (2 pp. per correct answer)

- - Approximately 1 in 1000 women taking the pill will experience a thrombosis per year (depending on the type of pill).
  - Approximately 50 in 1000 women taking the pill will experience a thrombosis per year (depending on the type of pill).
  - Approximately 100 in 1000 women taking the pill will experience a thrombosis per year (depending on the type of pill).
  - I don’t know

Part 3: Perceptions and feelings towards oral contraception (8 Questions)

**Q30** How much do you trust the information about oral contraceptives given to you by your gynecologist?

- - 100%
  - Enough.
  - Not in all respects
  - Not enough
  - Not at all.
  - No statement

**Q31** How many of your female friends are taking the pill?

- - Almost all of them
  - Probably more than 50%
  - Probably less than 50%
  - Only a few
  - I don’t know
  - No statement

**Q32** Have you experienced any changes in perception of the pill in your circle of friends and family?

- - I never speak to my friends or family about the pill.
  - No
  - Yes

->**Q33** How did the perception change in your circle of friends?

- - - - A positive perception of the pill is more common now.
      - A negative perception of the pill is more common now.
  - No statement

**Q34** About which aspects of the pill do you feel the least comfortable? *(Please choose one or two)*

- - Safety
  - Mild side effects (e.g. weight gain, headache, intermenstrual bleeding, etc)
  - Severe side effects (e.g. thrombosis)
  - Long term effects on risk of cancer
  - Long term effects on fertility
  - The environment
  - Other
  - No aspect at all
  - No statement

**Q35** How did your perception of the pill has changed since you first heard about it?

- - Not at all.
  - I’ve a more positive perception of the pill than I had before.
  - I’ve a more negative perception of the pill than I had before.
  - No statement

**Q36** If you stated a change in perception in the question before, what are the reasons for that change? *Select all that apply*

- - Own experience
  - Experience and stories shared by friends and family
  - Journalism and public media
  - Information found on the Internet
  - Information given to me by my gynecologist.
  - Other
  - No change stated
  - No statement

**Q37** Would you recommend your daughter to take the pill if she was in the appropriate age now?

- - No.
  - Yes.
  - I don’t know.
  - No statement

**Q38** What is the most important aspect for you when deciding against or for the pill?? *(Please choose one or two)*

- - Safety of birth control
  - Inconvenience with daily intake
  - Mild side effects
  - Severe side effects
  - Long term effects on risk of cancer
  - Long term effects on fertility
  - No aspect at all
  - No statement

Thank you very much for taking part in our study!

You can download the correct answers to the questions asked in the knowledge part below.

Space for your comments or feedback:
