# Supplementary material for: Survey of young women's state of knowledge and perceptions about oral contraceptives in Germany
Source: AJOG Glob Rep. 2022 Oct 7;2(4):100119. doi: 10.1016/j.xagr.2022.100119 (PMC9633744; doi:10.1016/j.xagr.2022.100119)
Supplement: Supplementary file 2 [file mmc2.docx]

Supplement B

B.1

**Sample Size**

There were not much information on potential results regarding the research questions. The estimated proportion of the population that presents with a particular attribute interesting for the research question is estimated to be 50% to allow for maximum variability. To calculate the sample size, the margin of error was set to 1.96 for a 95% confidence, and a minimum 5% precision.

$$384.16=\frac{{1.96}^{2} \left( 0.5 x 0.5 \right)}{{0.05}^{2}}$$

Thus, a random sample of 385 women was needed for this study.

B.2

In the survey, participants were asked to estimate their knowledge regarding three aspects of the pill: efficacy of contraception with the pill, the side effects, and the mode of action. The Total Subjective knowledge score were determined as the average of these three estimations. To enable an ordinal scale, the participants who answered one of the three questions “I don’t know” / “No statement” were excluded for accuracy estimation and left out for relevant analyses. Again, five levels of knowledge were achievable: Very high, high, centre, low or very low.
