# Supplementary material for: Survey of young women's state of knowledge and perceptions about oral contraceptives in Germany
Source: AJOG Glob Rep. 2022 Oct 7;2(4):100119. doi: 10.1016/j.xagr.2022.100119 (PMC9633744; doi:10.1016/j.xagr.2022.100119)
Supplement: Supplementary file 3 [file mmc3.docx]

Figure C.1: Attrition diagram: proportion of respondents remaining by Survey Question Number

Figure C.2: Demographical Data: Age
