# Supplementary material for: Survey of young women's state of knowledge and perceptions about oral contraceptives in Germany
Source: AJOG Glob Rep. 2022 Oct 7;2(4):100119. doi: 10.1016/j.xagr.2022.100119 (PMC9633744; doi:10.1016/j.xagr.2022.100119)
Supplement: Supplementary file 4 [file mmc4.docx]

| Subjective Knowledge | | | | | | | | | | |
| --- | --- | --- | --- | --- | --- | --- | --- | --- | --- | --- |
| By source of information | **Unadjusted** | | | | | **Adjusted for age** | | | | |
|  | b | (SE) | 95% CI for Odds Ratio | | | b | (SE) | 95% CI for Odds Ratio | |  |
| Very good vs. Not so good | | | Odds Ratio | Lower | Upper |  |  | Odds Ratio | Lower | Upper |
| Intercept | -1,80 | 0,24 |  |  |  | -2,52 | 0,80 |  |  |  |
| Gynecologist | 0,48* | 0,19 | 1,62 | 1,12 | 2,34 | 0,46* | 0,19 | 1,59 | 1,10 | 2,30 |
| Friends and Family | -0,33* | 0,16 | 0,72 | 0,53 | 0,98 | -0,31* | 0,16 | 0,73 | 0,54 | 1,00 |
| School / University | 1,41*** | 0,18 | 4,09 | 2,85 | 5,86 | 1,41*** | 0,19 | 4,11 | 2,86 | 5,90 |
| Internet and social media | -0,20 | 0,19 | 0,82 | 0,56 | 1,18 | -0,21 | 0,19 | 0,81 | 0,56 | 1,18 |
| Books and Papers | 0,51** | 0,17 | 1,67 | 1,20 | 2,33 | 0,50** | 0,17 | 1,65 | 1,18 | 2,31 |
| Good vs. Not so good | | | | | | | | | | |
| Intercept | -0,18 | 0,15 |  |  |  | -1,02 | 0,49 |  |  |  |
| Gynecologist | 0,51*** | 0,11 | 1,66 | 1,34 | 2,07 | 0,49*** | 0,11 | 1,64 | 1,31 | 2,04 |
| Friends and Family | -0,02 | 0,09 | 0,99 | 0,82 | 1,18 | 0,00 | 0,10 | 1,00 | 0,83 | 1,20 |
| School / University | 0,47** | 0,14 | 1,61 | 1,21 | 2,13 | 0,49** | 0,15 | 1,63 | 1,23 | 2,17 |
| Internet and social media | -0,10 | 0,12 | 0,91 | 0,72 | 1,15 | -0,09 | 0,12 | 0,91 | 0,72 | 1,15 |
| Books and Papers | 0,07 | 0,11 | 1,07 | 0,86 | 1,33 | 0,05 | 0,11 | 1,05 | 0,84 | 1,31 |
| Bad vs. Not so good | | | | | | | | | | |
| Intercept | -1,18 | 0,26 |  |  |  | -1,10 | 0,92 |  |  |  |
| Gynecologist | -0,44* | 0,19 | 0,65 | 0,44 | 0,94 | -0,46* | 0,19 | 0,63 | 0,43 | 0,92 |
| Friends and Family | -0,11 | 0,18 | 0,90 | 0,63 | 1,28 | -0,07 | 0,18 | 0,93 | 0,65 | 1,33 |
| School / University | -0,69 | 0,38 | 0,50 | 0,24 | 1,06 | -0,68 | 0,38 | 0,51 | 0,24 | 1,07 |
| Internet and social media | -0,38 | 0,23 | 0,69 | 0,44 | 1,07 | -0,38 | 0,23 | 0,68 | 0,43 | 1,07 |
| Books and Papers | 0,46* | 0,20 | 1,59 | 1,08 | 2,35 | 0,48* | 0,20 | 1,62 | 1,10 | 2,40 |
| Very bad vs. Not so good | | | | | | | | | | |
| Intercept | -2,24 | 0,58 |  |  |  | -5,45 | 2,27 |  |  |  |
| Gynecologist | -1,34** | 0,52 | 0,26 | 0,10 | 0,72 | -1,30* | 0,52 | 0,27 | 0,10 | 0,75 |
| Friends and Family | -0,51 | 0,54 | 0,60 | 0,21 | 1,73 | -0,43 | 0,55 | 0,65 | 0,22 | 1,89 |
| School / University | 0,52 | 0,66 | 1,69 | 0,46 | 6,13 | 0,58 | 0,66 | 1,79 | 0,49 | 6,50 |
| Internet and Social media | -1,16* | 0,56 | 0,31 | 0,10 | 0,94 | -1,04 | 0,56 | 0,35 | 0,12 | 1,07 |
| Books and Papers | 0,42 | 0,55 | 1,52 | 0,52 | 4,44 | 0,36 | 0,55 | 1,43 | 0,49 | 4,20 |

Supplement D

*Note. Unadjusted: R^2^ =0.06 (Cox-Snell). 0.07 (Nagelkerke). Model x^2^(20) = 150.57. p<0.001; Adjusted: R^2^ = 0.07 (Cox-Snell). 0.07 (Nagelkerke). Model x^2^(36) =165.72 ; p<0.001. *p < 0.05; **p < 0.01. ***p < 0.001.*

Table D.1: Subjective knowledge by Source of Information; Logistic regression, unadjusted and adjusted

| Knowledge test score by Source of information | | | | | | | |
| --- | --- | --- | --- | --- | --- | --- | --- |
| Unadjusted | | | | | | | |
| Source of information | Mean | SD | B | SE | Sig | F | Partial eta squared |
| Gynecologist | 17,9 | 3,37 | 0,62 | 0,16 | 0,00 | 14,86 | 0,01 |
| Others | 17,28 | 3,3 |  |  |  |  |  |
| Friends and Family | 17,77 | 3,34 | 0,03 | 0,14 | 0,86 | 0,03 | 0,00 |
| Others | 17,74 | 3,39 |  |  |  |  |  |
| School / University | 19,51 | 3,5 | 2,04 | 0,19 | 0,00 | 110,72 | 0,04 |
| Others | 17,48 | 3,26 |  |  |  |  |  |
| Internet and Social media | 17,91 | 3,32 | 0,75 | 0,17 | 0,00 | 20,07 | 0,01 |
| Others | 17,15 | 3,48 |  |  |  |  |  |
| Books and Papers | 18,17 | 3,54 | 0,54 | 0,16 | 0,00 | 11,11 | 0,00 |
| Others | 17,63 | 3,3 |  |  |  |  |  |
| Adjusted for age and educational background | | | | | | | |
| Source of information | Mean | SD | B | SE | Sig | F | Partial eta squared |
| Gynecologist | 17,9 | 3,37 | 0,60 | 0,16 | 0,00 | 14,20 | 0,01 |
| Others | 17,31 | 3,28 |  |  |  |  |  |
| Friends and Family | 17,77 | 3,33 | 0,07 | 0,14 | 0,60 | 0,28 | 0,00 |
| Others | 17,76 | 3,38 |  |  |  |  |  |
| School / University | 19,54 | 3,47 | 2,05 | 0,19 | 0,00 | 112,08 | 0,04 |
| Others | 17,49 | 3,25 |  |  |  |  |  |
| Internet and Social media | 17,91 | 3,32 | 0,81 | 0,17 | 0,00 | 23,08 | 0,01 |
| Others | 17,19 | 3,45 |  |  |  |  |  |
| Books and Papers | 18,19 | 3,52 | 0,50 | 0,16 | 0,00 | 9,78 | 0,00 |
| Others | 17,64 | 3,29 |  |  |  |  |  |

*Note. Levene’s Test of Equality of Error Variance F=1.152. p=0.26*

Table D.2: Knowledge test score by Source of Information; Univariate Logistic Regression, unadjusted and adjusted

| Accuracy of self-estimated knowledge | | | | | | | | | | |
| --- | --- | --- | --- | --- | --- | --- | --- | --- | --- | --- |
| By source of information | **Unadjusted** | | | | | **Adjusted for age and educational background** | | | | |
|  | b | (SE) | 95% CI for Odds Ratio | | | b | (SE) | 95% CI for Odds Ratio | | |
| Overestimation vs. Accurate estimation | | | Odds Ratio | Lower | Upper |  |  | Odds Ratio | Lower | Upper |
| Intercept | -0,10 | 0,15 |  |  |  | 0,55 | 0,51 |  |  |  |
| Gynecologist | -0,09 | 0,12 | 0,917 | 0,725 | 1,158 | -0,07 | 0,12 | 0,94 | 0,74 | 1,19 |
| Friends and Family | 0,11 | 0,10 | 1,117 | 0,919 | 1,356 | 0,10 | 0,10 | 1,10 | 0,91 | 1,34 |
| School / University | 0,04 | 0,14 | 1,044 | 0,793 | 1,374 | 0,04 | 0,14 | 1,04 | 0,79 | 1,37 |
| Internet and Social media | -0,42** | 0,12 | 0,66 | 0,521 | 0,836 | -0,41** | 0,12 | 0,66 | 0,52 | 0,84 |
| Books and Papers | 0,13 | 0,12 | 1,133 | 0,901 | 1,425 | 0,13 | 0,12 | 1,14 | 0,90 | 1,43 |
| Underestimation vs. Accurate estimation | | |  |  |  |  |  |  |  |  |
| Intercept | -0,48 | 0,17 |  |  |  | -0,27 | 0,56 |  |  |  |
| Gynecologist | -0,35** | 0,12 | 0,70 | 0,56 | 0,89 | -0,37** | 0,12 | 0,69 | 0,55 | 0,88 |
| Friends and Family | 0,00 | 0,11 | 1,00 | 0,81 | 1,23 | 0,00 | 0,11 | 1,00 | 0,81 | 1,23 |
| School / University | 0,09 | 0,15 | 1,09 | 0,81 | 1,46 | 0,10 | 0,15 | 1,10 | 0,82 | 1,48 |
| Internet and Social media | 0,06 | 0,14 | 1,07 | 0,81 | 1,41 | 0,05 | 0,14 | 1,05 | 0,80 | 1,39 |
| Books and Papers | 0,19 | 0,12 | 1,21 | 0,95 | 1,54 | 0,20 | 0,12 | 1,22 | 0,96 | 1,55 |

*Note. Unadjusted: R^2^ = 0.01 (Cox-Snell). 0.01 (Nagelkerke). Model x^2^(10) =30.21. p= 0.001; Adjusted: R^2^ =0.02 (Cox-Snell). 0.02 (Nagelkerke). Model x^2^(18) =38.74. p <0.01; *p < 0.05; **p < 0.01. ***p < 0.001.*

Table D.3: Accuracy of self-estimation by Source of Information; Logistic regression, unadjusted and adjusted

| Change in perception | | | | | | | | | | |
| --- | --- | --- | --- | --- | --- | --- | --- | --- | --- | --- |
| By source of information | **Unadjusted** | | | | | **Adjusted for age** | | | | |
|  | b | (SE) | 95% CI for Odds Ratio | | | b | (SE) | 95% CI for Odds Ratio | |  |
| I see the pill less critical vs No change | | | Odds Ratio | Lower | Upper |  |  | Odds Ratio | Lower | Upper |
| Intercept | -1,90 | 0,51 |  |  |  | 0,64 | 1,82 |  |  |  |
| Gynecologist | -0,45 | 0,41 | 0,64 | 0,29 | 1,43 | -0,40 | 0,41 | 0,67 | 0,30 | 1,50 |
| Friends and Family | 0,23 | 0,34 | 1,25 | 0,65 | 2,43 | 0,14 | 0,34 | 1,16 | 0,59 | 2,25 |
| School / University | -0,24 | 0,44 | 0,79 | 0,33 | 1,87 | -0,22 | 0,44 | 0,80 | 0,34 | 1,91 |
| Internet and Social media | 0,61 | 0,41 | 1,84 | 0,82 | 4,12 | 0,52 | 0,42 | 1,68 | 0,74 | 3,81 |
| Books and Papers | -0,92 | 0,50 | 0,40 | 0,15 | 1,06 | -0,89 | 0,50 | 0,41 | 0,15 | 1,10 |
| I see the pill more critical vs No change | | | | | | | | | | |
| Intercept | 1,88 | 0,20 |  |  |  | 0,16 | 0,67 |  |  |  |
| Gynecologist | -0,46** | 0,17 | 0,63 | 0,45 | 0,88 | -0,49** | 0,18 | 0,62 | 0,44 | 0,87 |
| Friends and Family | 0,12 | 0,13 | 1,13 | 0,87 | 1,46 | 0,15 | 0,13 | 1,17 | 0,90 | 1,51 |
| School / University | -0,62*** | 0,16 | 0,54 | 0,39 | 0,74 | -0,63*** | 0,16 | 0,54 | 0,39 | 0,74 |
| Internet and social media | 0,67*** | 0,15 | 1,96 | 1,47 | 2,61 | 0,70*** | 0,15 | 2,01 | 1,51 | 2,69 |
| Books and Papers | -0,03 | 0,16 | 0,97 | 0,72 | 1,32 | -0,06 | 0,16 | 0,94 | 0,70 | 1,28 |

*Note. Unadjusted: R^2^ = 0.02(Cox-Snell). 0.05 (Nagelkerke). Model x^2^(10) =56.19 p<0.001; Adjusted: R^2^ = 0.04 (Cox-Snell). 0.06 (Nagelkerke). Model x^2^(18) = 91.02 ; p<0.001.*p < 0.05; **p < 0.01. ***p < 0.001.*

Table D.4: Change in perception by Source of Information; Logistic regression, unadjusted and adjusted

| How much do you trust the information you’re getting from your Gynecologist | | | | | | | | | | |
| --- | --- | --- | --- | --- | --- | --- | --- | --- | --- | --- |
| By source of information | Unadjusted | | | | | Adjusted for age | | | | |
|  | b | (SE) | 95% CI for Odds Ratio | | | b | (SE) | 95% CI for Odds Ratio | |  |
| Completely vs. Not in all matters | | | Odds Ratio | Lower | Upper |  |  | Odds Ratio | Lower | Upper |
| Intercept | -1,57 | 0,27 |  |  |  | -0,27 | 0,78 |  |  |  |
| Gynecologist | 1,14*** | 0,25 | 3,13 | 1,94 | 5,06 | 1,15*** | 0,25 | 3,16 | 1,95 | 5,12 |
| Friends and Family | -0,49** | 0,15 | 0,61 | 0,46 | 0,83 | -0,56*** | 0,15 | 0,57 | 0,43 | 0,78 |
| School / University | 0,56** | 0,20 | 1,76 | 1,19 | 2,59 | 0,58** | 0,20 | 1,78 | 1,20 | 2,64 |
| Internet and social media | -0,74*** | 0,16 | 0,48 | 0,35 | 0,66 | -0,76*** | 0,17 | 0,47 | 0,34 | 0,65 |
| Books and Papers | -0,49* | 0,20 | 0,61 | 0,42 | 0,90 | -0,47* | 0,20 | 0,63 | 0,43 | 0,92 |
| Enough vs. Not in all matter | | | | | | | | | | |
| Intercept | -0,83 | 0,18 |  |  |  | -0,24 | 0,56 |  |  |  |
| Gynecologist | 0,89*** | 0,15 | 2,42 | 1,80 | 3,26 | 0,93*** | 0,15 | 2,53 | 1,87 | 3,41 |
| Friends and Family | -0,14 | 0,11 | 0,87 | 0,71 | 1,07 | -0,16 | 0,11 | 0,85 | 0,69 | 1,05 |
| School / University | 0,52*** | 0,15 | 1,67 | 1,26 | 2,23 | 0,49** | 0,15 | 1,63 | 1,22 | 2,18 |
| Internet and social media | -0,32* | 0,13 | 0,73 | 0,56 | 0,93 | -0,34* | 0,13 | 0,72 | 0,55 | 0,92 |
| Books and Papers | -0,41** | 0,13 | 0,67 | 0,52 | 0,86 | -0,40** | 0,13 | 0,67 | 0,52 | 0,87 |
| Not Enough vs. Not in all matter | | | | | | | | | | |
| Intercept | -0,63 | 0,20 |  |  |  | -1,38 | 0,64 |  |  |  |
| Gynecologist | -0,80*** | 0,13 | 0,45 | 0,35 | 0,58 | -0,81*** | 0,13 | 0,44 | 0,34 | 0,57 |
| Friends and Family | -0,07 | 0,12 | 0,93 | 0,73 | 1,19 | -0,05 | 0,13 | 0,95 | 0,75 | 1,22 |
| School / University | -0,02 | 0,19 | 0,99 | 0,68 | 1,43 | 0,00 | 0,19 | 1,00 | 0,69 | 1,46 |
| Internet and social media | 0,33 | 0,18 | 1,39 | 0,97 | 1,99 | 0,35 | 0,19 | 1,42 | 0,99 | 2,04 |
| Books and Papers | 0,09 | 0,14 | 1,09 | 0,83 | 1,43 | 0,07 | 0,14 | 1,08 | 0,82 | 1,41 |
| Not at all vs. Not in all matters | | | | | | | | | | |
| Intercept | -1,61 | 0,35 |  |  |  | -1,65 | 1,09 |  |  |  |
| Gynecologist | -1,58*** | 0,21 | 0,21 | 0,14 | 0,31 | -1,61*** | 0,21 | 0,20 | 0,13 | 0,30 |
| Friends and Family | -0,14 | 0,21 | 0,87 | 0,58 | 1,30 | -0,18 | 0,21 | 0,84 | 0,55 | 1,26 |
| School / University | 0,32 | 0,29 | 1,37 | 0,78 | 2,42 | 0,37 | 0,29 | 1,44 | 0,82 | 2,55 |
| Internet and social media | 0,34 | 0,34 | 1,40 | 0,72 | 2,74 | 0,34 | 0,34 | 1,40 | 0,72 | 2,75 |
| Books and Papers | 0,48* | 0,21 | 1,62 | 1,07 | 2,43 | 0,46* | 0,21 | 1,58 | 1,04 | 2,39 |

*Note. Unadjusted: R^2^ = 0.14(Cox-Snell).0.14 (Nagelkerke). Model x^2^(20) =354.26 p<0.001 ; Adjusted: R^2^ = 0.15 (Cox-Snell). 0.16 (Nagelkerke). Model x^2^(36) = 384.09 ; p<0.001. *p < 0.05; **p < 0.01. ***p < 0.001.*

Table D.5: Trust in Gynecologist by Source of Information; Logistic regression, unadjusted and adjusted

| Would you recommend the pill to your daughter? | | | | | | | | | | |
| --- | --- | --- | --- | --- | --- | --- | --- | --- | --- | --- |
| By source of information | **Unadjusted** | | | | | **Adjusted for age and educational background** | | | | |
|  | b | (SE) | 95% CI for Odds Ratio | | | b | (SE) | 95% CI for Odds Ratio | |  |
| Yes vs. I don't know | | | Odds Ratio | Lower | Upper |  |  | Odds Ratio | Lower | Upper |
| Intercept | -1,70 | 0,50 |  |  |  | -0,76 | 1,18 |  |  |  |
| Gynecologist | 1,77*** | 0,48 | 5,85 | 2,28 | 15,03 | 1,74*** | 0,48 | 5,70 | 2,21 | 14,68 |
| Friends and Family | -0,67** | 0,23 | 0,51 | 0,33 | 0,80 | -0,75** | 0,23 | 0,47 | 0,30 | 0,74 |
| School / University | 0,56 | 0,30 | 1,76 | 0,99 | 3,14 | 0,62* | 0,30 | 1,85 | 1,03 | 3,34 |
| Internet and Social media | -0,86*** | 0,24 | 0,42 | 0,27 | 0,67 | -0,93*** | 0,24 | 0,39 | 0,25 | 0,63 |
| Books and Papers | -0,52 | 0,34 | 0,59 | 0,30 | 1,16 | -0,51 | 0,35 | 0,60 | 0,31 | 1,18 |
| Rather yes vs. I don't know | | | | | | | | | | |
| Intercept | -0,20 | 0,25 |  |  |  | 1,58 | 0,78 |  |  |  |
| Gynecologist | 0,84*** | 0,21 | 2,33 | 1,56 | 3,47 | 0,81*** | 0,21 | 2,25 | 1,50 | 3,37 |
| Friends and Family | -0,34* | 0,15 | 0,71 | 0,53 | 0,95 | -0,40** | 0,15 | 0,67 | 0,50 | 0,90 |
| School / University | 0,50* | 0,21 | 1,66 | 1,10 | 2,49 | 0,59** | 0,21 | 1,80 | 1,19 | 2,72 |
| Internet and Social media | -0,04 | 0,18 | 0,96 | 0,67 | 1,37 | -0,11 | 0,18 | 0,90 | 0,63 | 1,29 |
| Books and Papers | -0,12 | 0,19 | 0,89 | 0,61 | 1,30 | -0,12 | 0,20 | 0,89 | 0,61 | 1,31 |
| Rather No vs. I don't know | | | | | | | | | | |
| Intercept | 0,99 | 0,21 |  |  |  | 0,77 | 0,70 |  |  |  |
| Gynecologist | -0,20 | 0,16 | 0,82 | 0,60 | 1,12 | -0,25 | 0,16 | 0,78 | 0,57 | 1,07 |
| Friends and Family | -0,20 | 0,13 | 0,82 | 0,63 | 1,07 | -0,20 | 0,14 | 0,82 | 0,63 | 1,07 |
| School / University | 0,01 | 0,20 | 1,01 | 0,68 | 1,50 | 0,06 | 0,20 | 1,06 | 0,71 | 1,58 |
| Internet and Social media | 0,08 | 0,17 | 1,09 | 0,78 | 1,52 | 0,05 | 0,17 | 1,05 | 0,75 | 1,48 |
| Books and Papers | 0,37* | 0,17 | 1,45 | 1,05 | 2,02 | 0,37* | 0,17 | 1,45 | 1,04 | 2,02 |
| No vs. I don't know | | | | | | | | | | |
| Intercept | 1,01 | 0,22 |  |  |  | 0,43 | 0,73 |  |  |  |
| Gynecologist | -0,62*** | 0,16 | 0,54 | 0,39 | 0,74 | -0,67*** | 0,16 | 0,51 | 0,37 | 0,70 |
| Friends and Family | -0,44** | 0,14 | 0,65 | 0,49 | 0,85 | -0,44** | 0,14 | 0,65 | 0,49 | 0,85 |
| School / University | 0,01 | 0,21 | 1,01 | 0,67 | 1,51 | 0,07 | 0,21 | 1,07 | 0,71 | 1,62 |
| Internet and Social media | 0,21 | 0,18 | 1,23 | 0,86 | 1,75 | 0,18 | 0,19 | 1,20 | 0,84 | 1,72 |
| Books and Papers | 0,76*** | 0,17 | 2,15 | 1,54 | 2,99 | 0,75*** | 0,17 | 2,12 | 1,52 | 2,96 |

*Note. Unadjusted: R^2^ = 0.10 (Cox-Snell). 0.11 (Nagelkerke). Model x^2^(20) = 264.978. p<0.001 ; Adjusted: R^2^ = 0.12 (Cox-Snell). 0.12 (Nagelkerke). Model x^2^(36) 306.44 ; p<0.001. *p < 0.05; **p < 0.01. ***p < 0.001.*

Table D.6: Recommendation of the pill to hypothetical daughter by Source of Information; Logistic regression, unadjusted and adjusted

| Change in perception | | | | | | | | | | |
| --- | --- | --- | --- | --- | --- | --- | --- | --- | --- | --- |
| By Knowledge test score | Unadjusted | | | | | Adjusted for age | | | | |
|  | b | (SE) | 95% CI for Odds Ratio | | | b | (SE) | 95% CI for Odds Ratio | | |
|  |  |  | Odds Ratio | Lower | Upper |  |  | Odds Ratio | Lower | Upper |
| less critical vs. No change | | | | | | | | | | |
| Intercept | -1.49 | 0,87 |  |  |  | 1,15 | 1,86 |  |  |  |
| Final score | -0.02 | 0,05 | 0,98 | 0,89 | 1,07 | -0,01 | 0,05 | 0,99 | 0,90 | 1,09 |
| More critical vs. No change | | | | | | | | | | |
| Intercept | 2.46 | 0,34 |  |  |  | 1,33 | 0,69 |  |  |  |
| Final score | -0.03 | 0,02 | 0,97 | 0,94 | 1,01 | -0,04* | 0,02 | 0,96 | 0,93 | 1,00 |

*Note. Unadjusted: R^2^ = 0.001 (Cox-Snell), 0.002 (Nagelkerke). Model x^2^(2) =2.28 ; p=0.32, Adjusted: R^2^ = 0.02 (Cox-Snell), 0.03(Nagelkerke). Model x^2^(10) =37.55 ; p<0.001.*p < 0.05; **p < 0.01. ***p < 0.001.*

Table D.7: Change in perception by Knowledge test score; Logistic regression, unadjusted and adjusted

| How much do you trust the information you’re getting from your Gynecologist? | | | | | | | | | | |
| --- | --- | --- | --- | --- | --- | --- | --- | --- | --- | --- |
| By Knowledge test score | Unadjusted | | | | | Adjusted for age | | | | |
|  | b | (SE) | 95% CI for Odds Ratio | | | b | (SE) | 95% CI for Odds Ratio | | |
|  |  |  | Odds Ratio | Lower | Upper |  |  | Odds Ratio | Lower | Upper |
| Completely vs. Not in all matters | | | | | | | | | | |
| Intercept | -2.95 | 0.39 |  |  |  | -2,31 | 0,78 |  |  |  |
| Final score | 0.09*** | 0.02 | 1,09 | 1,05 | 1,14 | 0,10*** | 0,02 | 1,10 | 1,06 | 1,15 |
| Enough vs. Not in all matters | | | | | | | | | | |
| Intercept | -1.42 | 0.28 |  |  |  | -1,03 | 0,56 |  |  |  |
| Final score | 0.06*** | 0.02 | 1,06 | 1,03 | 1,09 | 0,06*** | 0,02 | 1,06 | 1,03 | 1,10 |
| Not Enough vs. Not in all matters | | | | | | | | | | |
| Intercept | -0.80 | 0.31 |  |  |  | -1,45 | 0,64 |  |  |  |
| Final score | -0.01 | 0.02 | 0,99 | 0,96 | 1,03 | -0,01 | 0,02 | 0,99 | 0,96 | 1,03 |
| Not at all vs. Not in all matters | | | | | | | | | | |
| Intercept | -1.61 | 0.51 |  |  |  | -1,73 | 1,08 |  |  |  |
| Final score | -0.03 | 0.03 | 0,97 | 0,92 | 1,03 | -0,03 | 0,03 | 0,97 | 0,91 | 1,02 |

*Note. Unadjusted: R^2^ = 0.01 (Cox-Snell). 0.01 (Nagelkerke). Model x^2^(4) =33.17 ; p<0.001. Adjusted: R^2^ = 0.03 (Cox-Snell). 0.03 (Nagelkerke). Model x^2^(20) = 61.99; p<0.001.*p < 0.05; **p < 0.01. ***p < 0.001.*

Table D.8: Trust in Gynecologist by Knowledge test score; Logistic regression, unadjusted and adjusted

| Would you recommend the pill to your daughter? | | | | | | | | | | |
| --- | --- | --- | --- | --- | --- | --- | --- | --- | --- | --- |
| By Knowledge test score | Unadjusted | | | | | Adjusted for age and educational background | | | | |
|  | b | (SE) | 95% CI for Odds Ratio | | | b | (SE) | 95% CI for Odds Ratio | | |
|  |  |  | Odds Ratio | Lower | Upper |  |  | Odds Ratio | Lower | Upper |
| Yes vs. I don't know | | | | | | | | | | |
| Intercept | -1.93 | 0.57 |  |  |  | -1,90 | 1,15 |  |  |  |
| Final score | 0.05 | 0.03 | 1,05 | 0,99 | 1,12 | 0,04 | 0,03 | 1,05 | 0,98 | 1,11 |
| Rather yes vs. I don't know | | | | | | | | | | |
| Intercept | -0.93 | 0.39 |  |  |  | 0,64 | 0,79 |  |  |  |
| Final score | 0.07** | 0.02 | 1,08 | 1,03 | 1,12 | 0,07** | 0,02 | 1,07 | 1,02 | 1,11 |
| Rather No vs. I don't know | | | | | | | | | | |
| Intercept | 0.32 | 0.35 |  |  |  | 0,15 | 0,71 |  |  |  |
| Final score | 0.03 | 0.02 | 1,03 | 0,99 | 1,07 | 0,02 | 0,02 | 1,02 | 0,98 | 1,06 |
| No vs. I don't know | | | | | | | | | | |
| Intercept | 1.10 | 0.35 |  |  |  | 0,36 | 0,73 |  |  |  |
| Final score | -0.02 | 0.02 | 0,98 | 0,94 | 1,02 | -0,04 | 0,02 | 0,96 | 0,93 | 1,00 |

*Note. Unadjusted: R^2^ =0.01 (Cox-Snell), 0.01 (Nagelkerke). Model x^2^(4) = 32.90 ; p<0.001., Adjusted: R^2^ =0.03 (Cox-Snell), 0.03 (Nagelkerke). Model x^2^(20) = 71.66 ; p<0.001. *p < 0.05; **p < 0.01. ***p < 0.001.*

Table D.9: Recommendation to hypothetical daughter by Knowledge test score; Logistic regression, unadjusted and adjusted

| Change in perception | | | | | | | | | | |
| --- | --- | --- | --- | --- | --- | --- | --- | --- | --- | --- |
| By Subjective knowledge | Unadjusted | | | | | Adjusted for age | | | | |
|  | b | (SE) | 95% CI for Odds Ratio | | | b | (SE) | 95% CI for Odds Ratio | | |
|  |  |  | Odds Ratio | Lower | Upper |  |  | Odds Ratio | Lower | Upper |
| less critical vs. No change | | | | | | | | | | |
| Intercept | -0,29 | 0,76 |  |  |  | 2,42 | 1,90 |  |  |  |
| Very High | -2,03* | 0,88 | 0,13 | 0,02 | 0,73 | -2,00* | 0,88 | 0,14 | 0,02 | 0,76 |
| High | -1,70* | 0,79 | 0,18 | 0,04 | 0,87 | -1,67* | 0,80 | 0,19 | 0,04 | 0,90 |
| Centre | -1,19 | 0,84 | 0,30 | 0,06 | 1,57 | -1,20 | 0,84 | 0,30 | 0,06 | 1,57 |
| Very low / low |  |  |  |  |  |  |  |  |  |  |
| More critical vs. No change | | | | | | | | | | |
| Intercept | 3,73 | 0,51 |  |  |  | 2,14 | 0,82 |  |  |  |
| Very High | -2,72*** | 0,53 | 0,07 | 0,02 | 0,19 | -2,76** | 0,53 | 0,06 | 0,02 | 0,18 |
| High | -2,11*** | 0,51 | 0,12 | 0,04 | 0,33 | -2,15*** | 0,51 | 0,12 | 0,04 | 0,32 |
| Centre | -0,77 | 0,53 | 0,46 | 0,16 | 1,30 | -0,75 | 0,53 | 0,47 | 0,17 | 1,34 |
| Very low / low |  |  |  |  |  |  |  |  |  |  |

*Note. Unadjusted: R^2^ = 0.05 (Cox-Snell). 0.09 (Nagelkerke). Model x^2^(6) =133.83 ; p<0.001. Adjusted: R^2^ = 0.07 (Cox-Snell). 0.12(Nagelkerke). Model x^2^(14) = 174.94; p<0.001.*p < 0.05; **p < 0.01. ***p < 0.001.*

Table D.10: Change in perception by Subjective knowledge; Logistic regression, unadjusted and adjusted

| How much do you trust the information you’re getting from your Gynecologist? | | | | | | | | | | |
| --- | --- | --- | --- | --- | --- | --- | --- | --- | --- | --- |
| By Subjective knowledge | Unadjusted | | | | | Adjusted for age | | | | |
|  | b | (SE) | 95% CI for Odds Ratio | | | b | (SE) | 95% CI for Odds Ratio | | |
|  |  |  | Odds Ratio | Lower | Upper |  |  | Odds Ratio | Lower | Upper |
| Completely vs. Not in all matters | | | | | | | | | | |
| Intercept | -2,55 | 0,42 |  |  |  | -1,54 | 0,839 |  |  |  |
| Very High | 2,50*** | 0,46 | 12,23 | 4,96 | 30,14 | 2,51*** | 0,46 | 12,28 | 4,97 | 30,32 |
| High | 1,48** | 0,43 | 4,40 | 1,88 | 10,31 | 1,48** | 0,44 | 4,38 | 1,87 | 10,30 |
| Centre | 0,16 | 0,46 | 1,18 | 0,48 | 2,88 | 0,12 | 0,46 | 1,13 | 0,46 | 2,76 |
| Very low / low |  |  |  |  |  |  |  |  |  |  |
| Enough vs. Not in all matters | | | | | | | | | | |
| Intercept | -2,26 | 0,37 |  |  |  | -1,60 | 0,63 |  |  |  |
| Very High | 2,325*** | 0,41 | 10,23 | 4,58 | 22,85 | 2,31*** | 0,41 | 10,09 | 4,51 | 22,59 |
| High | 2,148*** | 0,38 | 8,57 | 4,09 | 17,98 | 2,14*** | 0,38 | 8,50 | 4,05 | 17,87 |
| Centre | 1,423*** | 0,38 | 4,15 | 1,96 | 8,78 | 1,39*** | 0,38 | 4,00 | 1,89 | 8,47 |
| Very low / low |  |  |  |  |  |  |  |  |  |  |
| Not Enough vs. Not in all matters | | | | | | | | | | |
| Intercept | -0,28 | 0,17 |  |  |  | -0,98 | 0,62 |  |  |  |
| Very High | -0,54 | 0,29 | 0,58 | 0,33 | 1,02 | -0,59* | 0,29 | 0,55 | 0,32 | 0,97 |
| High | -1,087*** | 0,20 | 0,34 | 0,23 | 0,50 | -1,14*** | 0,21 | 0,32 | 0,22 | 0,48 |
| Centre | -0,407* | 0,19 | 0,67 | 0,46 | 0,97 | -0,44* | 0,20 | 0,65 | 0,44 | 0,95 |
| Very low / low |  |  |  |  |  |  |  |  |  |  |
| Not at all vs. Not in all matters | | | | | | | | | | |
| Intercept | -1,30 | 0,25 |  |  |  | -1,46 | 1,03 |  |  |  |
| Very High | -0,15 | 0,38 | 0,86 | 0,41 | 1,80 | -0,22 | 0,38 | 0,81 | 0,38 | 1,70 |
| High | -1,543*** | 0,32 | 0,21 | 0,11 | 0,40 | -1,62*** | 0,32 | 0,20 | 0,11 | 0,37 |
| Centre | -0,722* | 0,29 | 0,49 | 0,28 | 0,85 | -0,77** | 0,29 | 0,46 | 0,27 | 0,81 |
| Very low / low |  |  |  |  |  |  |  |  |  |  |

*Note. Unadjusted: R^2^ = 0.13 (Cox-Snell). 0.14 (Nagelkerke). Model x^2^(12) =327.40 ; p<0.001. Adjusted: R^2^ = 0.14 (Cox-Snell). 0.15 (Nagelkerke). Model x^2^(28) = 358.39; p<0.001.*p < 0.05; **p < 0.01. ***p < 0.001.*

Table D.11: Trust in Gynecologist by Subjective knowledge; Logistic regression, unadjusted and adjusted

| Would you recommend the pill to your daughter? | | | | | | | | | | |
| --- | --- | --- | --- | --- | --- | --- | --- | --- | --- | --- |
| By Subjective knowledge | Unadjusted | | | | | Adjusted for age | | | | |
|  | b | (SE) | 95% CI for Odds Ratio | | | b | (SE) | 95% CI for Odds Ratio | | |
|  |  |  | Odds Ratio | Lower | Upper |  |  | Odds Ratio | Lower | Upper |
| Yes vs. I don't know | | | | | | | | | | |
| Intercept | -2,71 | 1,03 |  |  |  | -2,27 | 1,48 |  |  |  |
| Very High | 2,37* | 1,06 | 10,66 | 1,33 | 85,61 | 2,39* | 1,06 | 10,95 | 1,36 | 88,06 |
| High | 2,10* | 1,04 | 8,19 | 1,06 | 63,20 | 2,12* | 1,04 | 8,29 | 1,07 | 64,06 |
| Centre | 0,68 | 1,07 | 1,98 | 0,25 | 15,93 | 0,71 | 1,07 | 2,04 | 0,25 | 16,42 |
| Very low / low |  |  |  |  |  |  |  |  |  |  |
| Rather yes vs. I don't know | | | | | | | | | | |
| Intercept | -0,51 | 0,42 |  |  |  | 1,09 | 0,85 |  |  |  |
| Very High | 0,68 | 0,48 | 1,97 | 0,78 | 5,01 | 0,80 | 0,49 | 2,23 | 0,85 | 5,84 |
| High | 1,26** | 0,43 | 3,53 | 1,51 | 8,27 | 1,38** | 0,45 | 3,96 | 1,64 | 9,58 |
| Centre | 0,50 | 0,44 | 1,65 | 0,70 | 3,92 | 0,62 | 0,46 | 1,86 | 0,76 | 4,54 |
| Very low / low |  |  |  |  |  |  |  |  |  |  |
| Rather No vs. I don't know | | | | | | | | | | |
| Intercept | 1,34 | 0,29 |  |  |  | 1,12 | 0,71 |  |  |  |
| Very High | -0,91* | 0,36 | 0,40 | 0,20 | 0,81 | -0,93** | 0,36 | 0,40 | 0,20 | 0,80 |
| High | -0,40 | 0,31 | 0,67 | 0,37 | 1,22 | -0,42 | 0,31 | 0,66 | 0,36 | 1,20 |
| Centre | -0,39 | 0,31 | 0,68 | 0,37 | 1,24 | -0,38 | 0,31 | 0,68 | 0,37 | 1,25 |
| Very low / low |  |  |  |  |  |  |  |  |  |  |
| No vs. I don't know | | | | | | | | | | |
| Intercept | 1,81 | 0,28 |  |  |  | 1,01 | 0,73 |  |  |  |
| Very High | -1,28*** | 0,35 | 0,28 | 0,14 | 0,55 | -1,29*** | 0,35 | 0,28 | 0,14 | 0,54 |
| High | -1,36*** | 0,30 | 0,26 | 0,14 | 0,46 | -1,38*** | 0,30 | 0,25 | 0,14 | 0,45 |
| Centre | -0,98** | 0,30 | 0,38 | 0,21 | 0,68 | -0,95** | 0,30 | 0,39 | 0,22 | 0,69 |
| Very low / low |  |  |  |  |  |  |  |  |  |  |

*Note. Unadjusted: R^2^ = 0.08 (Cox-Snell). 0.09 (Nagelkerke). Model x^2^(12) =205.26 ; p<0.001., Adjusted: R^2^ = 0.1 (Cox-Snell). 0.1(Nagelkerke). Model x^2^(28) = 249.00; p<0.001.*p < 0.05; **p < 0.01. ***p < 0.001.*

Table D.12: Recommendation to hypothetical daughter by Subjective Knowledge; Logistic regression, unadjusted and adjusted
