# Supplementary material for: Survey of young women's state of knowledge and perceptions about oral contraceptives in Germany
Source: AJOG Glob Rep. 2022 Oct 7;2(4):100119. doi: 10.1016/j.xagr.2022.100119 (PMC9633744; doi:10.1016/j.xagr.2022.100119)
Supplement: Supplementary file 5 [file mmc5.docx]

| Oral contraceptive history | Frequency | Percent |
| --- | --- | --- |
| Have you ever been taking the pill? | | |
| Yes | 2290 | 92,7 |
| No | 176 | 7,1 |
| No statement | 4 | 0,2 |
| Total | 2470 | 100 |
| Are you still taking the pill? | | |
| No. | 1480 | 64.5 |
| Yes. | 811 | 35,4 |
| No statement. | 3 | 0,1 |
| Total | 2294 | 100 |
| I am currently still taking the pill … |  |  |
| … and am very satisfied with it. | 159 | 19 |
| … and am satisfied with it. | 329 | 40 |
| … and am not satisfied / or currently thinking about stopping / changing to another method. | 321 | 40 |
| No statement. | 5 | 1 |
| Total | 811 | 100 |

Supplement E

E.1: Data on history with oral contraceptives

| Would you like to be better informed about the pill? | Frequency | Percent |
| --- | --- | --- |
| Yes | 1659 | 67,2 |
| No | 456 | 18,5 |
| I don’t know | 298 | 12,1 |
| No statement | 57 | 2,3 |
| Total | 2470 | 100 |

E.2: Need for information

|  | How well do you feel informed about the mode of action of oral contraception? | | How well do you feel informed about the safety of oral contraceptives? | | How well do you feel informed about the side effects caused by oral contraceptives? | |
| --- | --- | --- | --- | --- | --- | --- |
|  | Frequency | Percent | Frequency | Percent | Frequency | Percent |
| Very well. | 368 | 15% | 900 | 36% | 215 | 9% |
| Well. | 1199 | 49% | 1297 | 53% | 536 | 22% |
| Not so good. | 651 | 26% | 212 | 9% | 743 | 30% |
| Bad. | 157 | 6% | 26 | 1% | 492 | 20% |
| Very bad. | 67 | 3% | 16 | 1% | 475 | 19% |
| I don't know | 24 | 1% | 12 | 0% | 8 | 0% |
| No statement. | 4 | 0% | 7 | 0% | 1 | 0% |
| Total | 2470 | 100% | 2470 | 100% | 2470 | 100% |

E.3: Subjective knowledge for different aspects; frequency and proportion

| Would you recommend your daughter to take the pill if she was in the appropriate age now? | | |
| --- | --- | --- |
| Yes. | 126E | 5% |
| Rather yes. | 488 | 20% |
| Rather no. | 816 | 33% |
| No. | 687 | 28% |
| I don't know. | 340 | 14% |
| No statement. | 13 | 1% |
| Total. | 2470 | 100% |

E.4: Recommendation to hypothetical daughter

| The aspects with the highest level of uncertainty regarding the pill *(multi answer)* | Frequency | Percent |
| --- | --- | --- |
| fertility | 1246 | 50,4 |
| Common side effects | 1997 | 48,5 |
| Long-term effects on cancer risk | 984 | 39,8 |
| Rare side effects | 825 | 33,4 |
| Environmental impact of the pill | 244 | 9,9 |
| Safety of birth control | 105 | 4,3 |
| No statement | 27 | 1,1 |
| Total | 5428 | 187,4 |

E.5: The aspect with the highest level of uncertainty
